# Glasgow Royal Infirmary

**Published:** 1832-09

**Authors:** 


### GLASGOW ROYAL INFIRMARY.

### *Cases of Aneurism by Anastomosing, treated by* JOHN MACFARLANE, M.D.*

#### *Aneurism by Anastomosis; Tumor Pendulous; cured by temporary Ligature.*

A CHILD, eight months old, was brought to the Infirmary to have a pyriform tumor removed from the edge of the under lip. It was, when observed at birth, about the size of a split pea, of a livid colour, and on a level with the surrounding integuments. It remained stationary for the first three months, after which time it began to increase rapidly, and to project in a pendulous form. The integuments were entire: the apex of the tumor, which was about the size of a walnut, was irregular and doughy; whilst the neck, which was not larger than a quill, was hard and smooth, and the pulsations of its vessels were distinctly perceptible. A broad ligature of tape was firmly applied, close to the base of the tumor, and removed in twenty-four hours. I did not intend to allow the ligature to remain till ulceration of the pedicle was produced, but only, by obstructing the circulation for a few hours, to cause coagulation of the blood, and in this way to attempt to destroy the vitality of the tumor. It was, with the use of cold, perfectly successful: the tumor sphacelated, and the portion of lip to which it was attached speedily cicatrized.

I was induced to try the temporary use of the ligature, from having met with another case of this disease, in which, by the usual mode of its application, violent convulsions were produced.

#### *Aneurism by Anastomosis: Ligature of the Tumor; Convulsions; Cure.*

A child, nine months old, had a tumor, about the size of a grape, over the anterior superior angle of the left parietal bone, which had all the characters of aneurism by anastomosis. A needle, armed with a double ligature, was passed under its base, and each half of the swelling was tightly tied, so as to cut off its supply of blood. The child was teething: it cried bitterly, and was fretful and uneasy for several

* Macfarlane's Clinical Reports.

hours. During the following night it had an attack of convulsions, which continued for fifteen minutes, and returned with undiminished violence after an interval of two hours. The ligature was immediately removed, and the convulsions ceased. In four days the tumor sloughed, and a cure was speedily accomplished.

*Aneurism by Anastomosis; Ligature of the Tumor followed by Hemorrhage; cured by actual Cautery.*

W. H., aged seven months, had a soft, unequal, purple-coloured tumor, about the size of a half-a-crown, on the anterior surface of the left arm, two inches above the elbow-joint. It was elevated a little above the surrounding parts; and when firmly compressed, an obscure thrilling, or slightly pulsatory sensation, was perceptible. It was tied with a double ligature, as in the last case, and in six days the tumor separated. The exposed surface, which had at first a sloughy appearance, soon became clean and florid; and, except a small spot in the centre, it was evident that the diseased structure was completely destroyed. Here, however, a large spongy tumor formed, which was of a dark colour, and bled profusely. Pressure, by means of a compress and bandage, the free application of nitric acid, caustic, &c., were ineffectual in checking its progress. The actual cautery was at length had recourse to; and by four applications of it, the morbid growth was destroyed, and a cure accomplished.

In this case, it is probable, although the ligatures were carefully introduced under the base of the tumor, and firmly tied, that the whole of the diseased mass was not included. Even when this does happen, it is seldom that the disease is reproduced, as the enlarged vessels on which it depends become obliterated to some distance below the point at which the ligature is applied.

Whatever is capable of exciting inflammation in these vascular tumors, and of producing either ulceration or consolidation of their loose texture by the effusion of lymph, may put a stop to their progress, and ultimately lead to a cure. For this purpose I have used vaccination with success in five cases; and in one case, where the disease extended over the whole surface of the lower eyelid, and where neither the ligature nor the knife could be employed without producing deformity, I succeeded in exciting inflammation of the tumor, by introducing a seton close to its base, and retaining it till partial suppuration was established. In another case, where the disease was confined to the inside of the lower lip, the seton proved unsuccessful, and ligatures had to be employed.

